# Predicting Acute Ischaemic Stroke Outcome Using Clinical and Temporal Thresholds

**DOI:** 10.5402/2011/354642

**Published:** 2011-06-14

**Authors:** Denis Sablot, Faouzi Belahsen, Fabrice Vuillier, Jean-François Cassarini, Pierre Decavel, Laurent Tatu, Thierry Moulin, Elisabeth Medeiros de Bustos

**Affiliations:** ^1^Department of Neurology, Saint Jean Hospital, 20 Avenue du Languedoc, 66046 Perpignan Cedex, France; ^2^Department of Neurology, CHU Hassan II, Dhar Mehraz, Fes 30000, Morocco; ^3^Department of Neurology, CHU Besancon, 3 Boulevard Fleming, 25030 Besancon Cedex, France

## Abstract

*Background.* Few studies have analysed the natural course of cerebral ischaemia for predicting outcome. We aimed to determine the early clinical findings and the thresholds for deficit severity and symptom duration that make it possible to stratify outcome. *Methods.* We included 154 patients with transient ischaemic attack or ischaemic stroke. Stroke profiles and neurological status were assessed from onset to 24 hrs, on admission, at 48 hrs, and at discharge. Outcomes were evaluated using the modified Rankin Scale. Positive and negative predictive values were calculated for the different thresholds. The model was subsequently evaluated on a new prospective cohort of 157 patients. *Results.* Initial National Institute of Health Stroke Scale (NIHSS) score <5 and symptoms regressing within 135 min were predictive of good outcome. Initial NIHSS score >22 and symptom stability after 1,230 min were predictive of physical dependency or death. *Conclusions.* Low and high NIHSS cut-off points are effective positive predictive values for good and poor outcomes. Thresholds for symptom duration are less conclusive.

## 1. Introduction

The majority of studies on the prehospitalisation phase of cerebral ischaemia (ischaemic stroke [IS] and transient ischaemic attack [TIA]) have largely been conducted by emergency medical personnel [1–3] and have primarily focused on technical considerations, general practitioners' (GP) knowledge of cerebrovascular events, the time lapses before medical care is sought, and the measures needed to shorten these delays. 

However, the dynamic nature of cerebral ischaemia has been emphasised [4], and the process—in terms of time and space—involved in the transition from reversible ischaemia to irreversible infarction is not a uniform one. This may explain the extremely variable outcomes for any given trunk occlusion, including the “spectacular shrinking deficit” described by Kraemer et al. [5]. Evidence suggests that management of IS patients is not always adapted to individual pathophysiological states and that the natural course of IS needs to be assessed with an array of simple and available tests. Rapid assessment is all the more crucial given that thrombolytic treatment is highly time dependent (<3 hours after symptom onset). Although the management of cerebral ischaemia is considerably helped by neuroimaging techniques such as diffusion-perfusion MR imaging (DWI-PWI), these techniques cannot replace clinical findings such as symptom intensity and duration. Identifying the initial course and thresholds may constitute a predictive diagnostic tool and lead to better acute management of stroke. Indeed placebo group analyses from pivot studies have demonstrated a good reliability between baseline National Institutes of Health Stroke Scale (NIHSS) scores on admission and functional outcome or hospital disposition after stroke [6–8]. To our knowledge, only one recent study has specifically analysed the natural course of cerebral ischaemia to predict outcome [9].

The aims of this study were firstly to determine the early clinical findings that are predictive of outcome in acute stoke, and secondly, to establish the thresholds for deficit severity and symptom duration that make it possible to stratify outcome.

## 2. Patients and Methods

### 2.1. Study Population

The study had a prospective, observational cohort design. From September 2000 to March 2001, patients admitted to Besançon University Hospital within 24 hours of symptom onset with clinical and neuroimaging patterns of IS or TIA were screened for inclusion in the study (*n* = 361). We selected arterial ischaemia and excluded cerebral venous thrombosis and intracerebral haematoma or patients who had previously had a stroke and were dependent (corresponding to a modified Rankin Scale (mRS) score of 3). Patients were excluded as follows: 98 were admitted without consulting a GP, 27 had intraparenchymatous haematoma, 4 had cerebral venous thrombosis, 24 IS patients were admitted >24 hours after symptom onset and two were receiving thrombolytic therapy. Among the 206 IS patients admitted within <24 hours, 52 were excluded when the precise course of their symptoms could not be obtained (*n* = 32), because the GP could not be contacted or could not establish relevant and detailed clinical patterns (*n* = 18), or when the patient showed symptoms of events similar to IS (epilepsy, migraine) which could cast doubt on their diagnosis (2 cases). The remaining 154 eligible patients were assessed according to the Besançon Stroke Registry criteria, reported in greater detail elsewhere [10]. TIA was defined according to a symptom duration of <24 hours (pre-2004 definition) [11]. For the purposes of this study, posterior and anterior circulation strokes were not differentiated, and side of deficit was not specified.

### 2.2. Scoring and Questionnaire

Two scores were used to assess prehospitalisation clinical status. The first, termed the “Neurological Dysfunction Score” (NDS), reflected the patient's and/or his/her family's evaluation of the symptoms and their variation prior to assessment on admission (Table 3). This initial NDS, termed “NDS-0,” defined the initial onset time of the symptoms. The second score was the NIHSS retrospectively estimated by the GP, termed “NIHSS-GP.” For patients who had been referred by a GP, NIHSS-GP was performed as soon as possible (up to 12 hours) after admission by a phone interview with the GP using a standard procedure. Upon admission, the in-hospital clinical examination was carried out to establish a new NDS (NDS-1) and an admission NIHSS (NIHSS-1). In order to monitor stroke course and to estimate the maximal duration of symptoms within 24 hours, a further examination took place every 8 hours during the first day of hospitalisation (NIHSS-1a, NIHSS-1b), 24 hours after admission (NIHSS-2), and finally at discharge (NIHSS-3). The latter score was associated with the mRS. These neurological examinations were all performed by neurologists with NIHSS certification. 

The NDS was used to assess the variation of the deficit before admission, to identify the degree of variance in the course of IS, and to ensure relevance and agreement in score evolution (NIHSS and NDS). Several NIHSS items were unknown or insufficiently assessed by the GP. This was most frequent for the following items: dysarthria (21 patients), neglect (11 patients), visual loss (9 patients), and ataxia (8 patients). For these items, the NIHSS-GP item was considered to have the same value as the corresponding NIHSS-1 item if the NDS was stable during the same period. This score was disregarded if the NDS was unstable during the same period.

### 2.3. End Points

**Figure 1:**
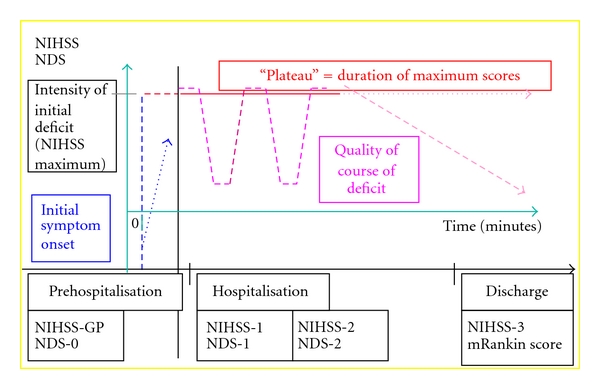
Analysis of IS course data.

The main data concerning the course of IS are shown in Figure 1. These relate to initial onset of symptoms (evaluated by NDS), intensity of initial deficit (evaluated by NIHSS-GP), duration of maximal scores (“plateau” evaluated by patient's and/or relatives' anamnesis and controlled by the NDS), course of deficit (evaluated by NIHSS and NDS score changes), maximum NIHSS (out of NIHSS-GP, NIHSS-1, NIHSS-1a, NIHSS-1b, and NIHSS-2) during the first 24 hours (“NIHSS-max”), and maximum NDS (“NDS-max”). Threshold outcomes were established based on the symptom duration plateau corresponding to the average plateau duration estimated by the patient and/or his/her family once the symptoms had regressed, and by NIHSS-max for the intensity of signs corresponding to the average NIHSS-max score in the cohort. A worsening of neurological deficit was defined as an increase of 4+ points on the NIHSS and 2+ on the NDS. Conversely, a decrease of 4+ points on the NIHSS and 2+ points on the NDS represented a regression. Neurological deficit was considered stable for variations below 4 and 2 points on the NIHSS and the NDS respectively.

### 2.4. Statistical Analysis

Statistical analysis was carried out using SPSS software, version 13.0 (SPSS Inc, 2005). The Chi-square test, the Student's *t*-test, linear correlation, variance analysis and ROC curves were used to determine outcome thresholds. The results were significant if *P* < 0.05. Outcome was considered poor if patients died or were dependent (i.e., a score of 4-5 on the mRS) or good if patients were left with slight or no disability (mRS 0-1). 

Thresholds for NIHSS and deficit duration were calculated using ROC curves. The cut-offs corresponded to the point of the ROC curve where the tangent to the slope (*S*) was equal to *S* = ((1 − *p*)/*p*) · Δ, where *p* represents the prevalence of the group considered as positive according to the Besançon Stroke Registry data and Δ represents the “error overcost” due to false positive (FP) over that due to false negative (FN), in comparison to real positive (RP) and real negative (RN) diagnoses. Δ was calculated using the following formula: Δ = (cost FP-cost RN)/(cost FNcost RP). Ratio Δ was fixed at 2 in order to increase the positive predictive value (PPV) (reliability of the prognosis for the family), and when thrombolysis was considered, the risk of haemorrhagic transformation when treating by excess, over the benefit lost when not treating by default. The relative risks used were the same as those from the meta-analysis of results obtained by Hacke et al. [12]. 

PPV and negative predictive value (NPV) thresholds were calculated using the following formulae: PPV = *p* · Se/*p* · Se + (1 − *p*)·(1 − Sp), and NPV = (1 − *p*) · Sp/(1 − *p*) · Sp + *p*(1 − Se) where *p* represents the prevalence of the positive-considered group in the cohort. Se and Sp represent the sensitivity and specificity of the predefined threshold. 

The model was subsequently evaluated on a new and prospective cohort of 157 patients to validate the sensitivity and specificity of the different thresholds.

## 3. Results

### 3.1. Baseline Characteristics (Table 1)

**Figure 2:**
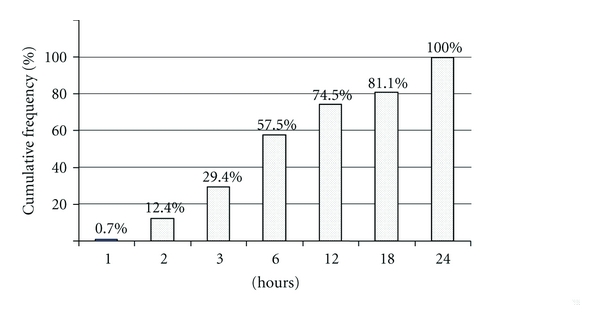
Onset to admission delays.

The study population comprised 154 patients including 88 men and 66 women (sex ratio 1.33). The mean age was 69.7 years ± 15.6 (22–96 years). In 36 (23%) patients, the neurological deficit regressed within 24 hours. In 26 (17%), the clinical presentation suggested an anterior circulatory system TIA and in the other 10 (6.5%) a TIA in the posterior circulatory system. Onset to admission times are outlined in Figure 2. Selected patients were all admitted within 24 hours of symptom onset, <3 hours for 45 (29.4%) patients and <6 hours for 89 (57.5%).

### 3.2. Initial Clinical Course

Symptom onset was sudden in 116 (75.3%) patients, progressive in 14 (9.2%), and unknown in 24 (15.5%). Clinical course during the first 24 hours was monophasic in 134 (87%) patients. This represented a stable initial deficit in 62 (40.2%), a total regression of symptoms in 36 (23.4%), and a partial regression in 36 (23.4%). In 20 (13%) patients, the initial presentation was polyphasic, that is, fluctuating in 10 (6.5%) and progressive in 10 (6.5%).

### 3.3. Correlation between NDS, NIHSS-1 and NIHSS-GP Scores (Figure 3)

There was a good correlation between the scores obtained on admission for NDS and NIHSS-1 (*r* ^2^ = 0.87). This was also the case for NIHSS-GP and NIHSS-1 scores on admission (*r* ^2^ = 0.79); mean time between the two physical examinations was 610.7 minutes.

### 3.4. Influence of Symptom Duration and Intensity on the Course of IS and Outcomes (Table 2)

Of the 36 TIA patients, 8 (22.2%) showed changes in their follow-up imaging studies (3 MRI and 5 CT-scan). However, only 12/28 (42.8%) patients with lesion-free TIA underwent MRI. The severity of neurological deficit, identified by NIHSS-max or NDS-max scores and deficit plateau, was correlated with the type of ischaemia (lesion-free TIA, TIA with lesion, stroke), but no significant differences were revealed between lesion-free TIA and TIA with lesion. In addition, initial deficit severity and plateau duration were strongly correlated with outcome after hospitalisation (Table 2). 

### 3.5. Factors Predictive of Outcome

In 150 (97.2%) patients (*χ* ^2^ = 44.1; *P* < 0.0001), a regression of the initial neurological signs within the first 24 hours was associated with a good prognosis (mRS 0-3). However in 92 (59.7%) patients (*χ* ^2^ = 49.2; *P* < 0.0001), a stable condition was significantly correlated with a negative prognosis (mRS 4-5 or death). Nevertheless, there was no significant relationship between the mRS and fluctuating (*χ* ^2^ = 2.5; *P* < 0.11) or progressive courses (*χ* ^2^ = 1.2; *P* < 0.45) within the first 24 hours.

### 3.6. Clinical and Temporal Thresholds Predicting Poor and Good Outcome (Figure 4)

**Figure 4:**
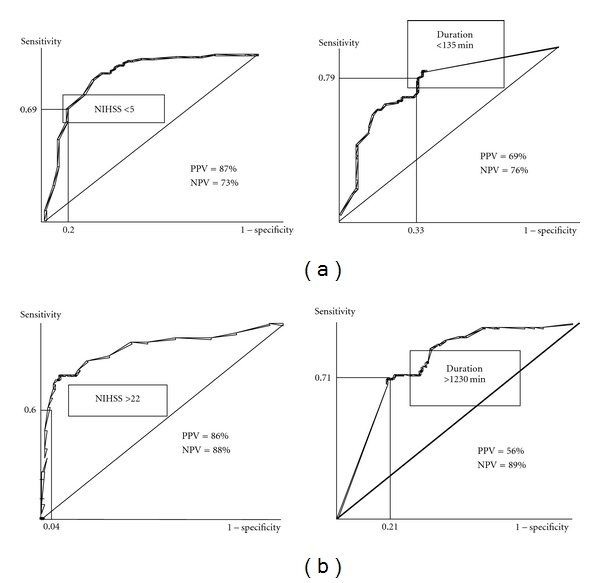
ROC curves calculating positive predictive value and negative predictive value. 4(a) For the prediction of a good outcome (mRS 0-1) for predefined clinical (left curve) and temporal (right curve) thresholds. 4(b) For the prediction of a poor outcome (mRS >3) for predefined clinical (left curve) and temporal (right curve) thresholds.

An NIHSS score >22 with a PPV of 86% and a NPV of 88% (Figure 4(a), left) was predictive of functional dependence (mRS 4-5) or death. The plateau duration threshold was 1,230 minutes (PPV = 56% and NPV = 89%) (Figure 4(a), right). An NIHSS score <5 was predictive of good functional outcome with a PPV of 86% and an NPV of 73% (Figure 4(b), left). The plateau duration threshold was 135 minutes (PPV = 69% and NPV = 76%) (Figure 4(b), right).

### 3.7. Model Evaluation

This model was evaluated and applied to a new prospective cohort of 157 patients. Good outcome prediction (mRS 0-1) showed 91% sensitivity and 88% specificity for NIHSS <5 and 100% sensitivity and 51% specificity for a plateau duration of <135 min. For NIHSS >22, the prediction of poor outcome (mRS >3) indicated 95% sensitivity and 85% specificity, and 67% sensitivity and 100% specificity for plateau duration >1,230 min.

## 4. Discussion

Although clinicians managing acute IS benefit from novel therapeutic approaches and better radiological evaluation, there is a lack of quantitative clinical criteria to enable IS to be stratified according to the vital and functional risks it generates. This lack of criteria is the result of rigid and uniform management of IS patients. The highly unpredictable outcome for any given arterial occlusion argues for a more individualised approach to the dynamic nature of ischaemia. Specific clinical criteria and temporal thresholds are more variable than neuroimaging data, even though certain models predicting outcome have recently been reported [13–16]. These models include neuroradiological variables and clinical events after admission, which precludes their use in decision-making on admission. Statistical models that predict functional outcome after stroke using 6 simple variables may prove useful in epidemiological studies, but until their impact on patient care and outcome has been evaluated, they should not be applied to clinical management [17]. A study of the course of IS in the first 24 hours and identification of the factors predictive of outcome could clarify nontreated patients' evolution using initial clinical data on admission. 

Previous studies have been based on an “a priori” model in which specific criteria were used to select patients [12]. In contrast, the homogeneity and reproducibility of our results stem from the fact that our study was based on an “a posteriori” model involving two consecutive, prospective cohorts who were all admitted in a routine setting. Covariables that predict outcome such as age, comorbidity, lesion size, and penumbra were not included for the statistical power of the tests and to ensure a practical application of the thresholds to initial clinical management. 

Despite the good reproducibility of the NIHSS [18], a bias may have been introduced by the subjectivity of each examiner (GPs for NIHSS-GP, neurologist for NIHSS-1). Moreover, even if some GPs had undergone initial training in the NIHSS, most of them did not use it regularly. To limit this bias, we excluded patients when the precise course of symptoms could not be ascertained (32 patients; 20.8%) or when GPs could not provide relevant and detailed clinical data (18 patients; 11.7%). However, data for the NIHSS-GP items were obtained for all selected patients, except for disregarded items (dysarthria, neglect, visual loss, and ataxia). This type of retrospective NIHSS scoring has already been validated with similar algorithms elsewhere [18, 19] with excellent reliability (resp. *r* ^2^ = 0.94, *P* < 0.001 and 86% probability of correctly ranking NIHSS). Using a similar methodological algorithm to perform NIHSS-GP, a good correlation between NIHSS-GP and NIHSS-1 (*r* ^2^ = 0.79) was observed even when the two scores were 610 minutes apart. We feel that reliability is improved by our method in which unknown NIHSS-GP items were either considered as equal to the corresponding NIHSS-1 or coded as unknown and not taken into account. Furthermore, in order to limit the risk of obtaining incomplete information from the GP, the patient, or his/her relatives, the history was taken as soon as possible after admission. We also performed a double analysis of the NIHSS and NDS scores from the patient's perspective. The NDS was used to evaluate the variation of symptoms before admission. This introduced a further possible source of bias, particularly due to the lack of objectivity and precision of the items (side, evaluation of cognitive disturbance, etc.), but it allowed us to evaluate the coherence of the temporal course of the initial symptoms and to ensure relevance and agreement in score evolution. The good correlations between NIHSS-GP and NIHSS-1 and between NIHSS-1 and NDS (*r* ^2^ = 0.87) both argue for the coherence of the different results and therefore for a limited bias. 

Deficit duration in TIA has already been widely discussed in the literature. As far back as 1983, Waxman and Toole [20] reported on TIA patients with CT scans revealing cerebral infarction. It was later established that evidence of recent infarction on cerebral imaging (CT scan or MRI) was directly correlated with symptom duration [21]. In 1999, two definitions of TIA were proposed according to whether symptoms lasted less or more than 1 hour [22]. Since 2004, the duration threshold has been reduced from 24 hours to 60 minutes. In our sample of patients with TIA, 23.4% had an event as defined before 2004, which is higher than in other comparable studies in the literature. This is probably due to the way in which medical care is organised in our hospital's emergency room [23] and also due to the low number of exclusions made possible by our ability to access temporal data with TIA. Among our TIA patients, test imaging study data were modified in 22.2% of cases, despite the low number of MRI studies performed (42.8%). Deficit severity and duration were on average higher in lesion-free TIA and lower in IS. Unlike Kimura et al. [22], there was no significant difference in symptom duration between lesion-free and nonlesion-free TIA. This is probably due to the lack of statistical power related to the small number of TIA patients with lesion. This was true when the mean duration of symptoms exceeded 7 hours (442 minutes), and the mean duration of lesion-free TIA deficit (75.2 minutes) was close to the 1 hour found by Kimura et al. [22]. 

The severity of neurological deficit, expressed by the initial NIHSS, predicted the functional prognosis at the end of hospitalisation: average initial NIHSS of 5.5 for nondependent, 17 for dependent, and 24.1 for deceased patients. Correlations between the severity of initial clinical presentation and a poor prognosis have been reported in only a few studies [24–28]. In these studies, progressive stroke also has a poor prognosis, but with a progression time definition subject to high variations (from <6 hours to 8 days) [26]. Moreover, DeGraba et al. [25] studied the criteria predictive of negative prognosis and worsening of the initial deficit in IS. According to these authors, an NIHSS score >7 represents a negative threshold in terms of worsening and functional outcome using a qualitative variable.

In our study the duration of neurological deficit was also a predictive criterion of functional outcome at discharge. On average, the plateau duration in our sample was >6 hours (399.1 minutes) for nondependent patients, >15 hours (931.5 minutes) for dependent patients, and close to 24 hours (1,230 minutes) for patients who died. Although these results appear evident in clinical practice, there is a dearth of precise data relating to the duration of the initial neurological deficit in the literature. 

The symptom thresholds that we defined met the selection requirements of patients for whom a therapeutic decision was likely to be considered. Here, two opposing points must be considered. The first corresponds to all patients who are likely to be dependent or dead at discharge; the prognosis threshold has a therapeutic implication (serious therapeutic decision) and implications for the family (negative prognosis announcement, application for nursing home care). Therefore, a threshold value with a maximum PPV is required. However, the temporal criteria threshold obtained was 1,230 minutes, with a PPV of 56% and an NPV of 89%. This criterion does not appear to accurately predict poor outcome since it was too long (close to 21 hours), and it was negative in nearly 50% of cases. Moreover, a regression of symptoms before this maximum threshold may also be predictive of an absence of functional dependence or death (NPV in 89% of cases). In the same way, an initial NIHSS score >22 was a better predictor of poor prognosis with a PPV of 86% and an NPV of 88%. This threshold would seem to more accurately define those patients who are more likely to have a poor outcome, even if it fails in about 10% of cases. Secondly, although recent studies indicate that patients with mild but disabling symptoms could be treated with tPA regardless of their baseline NIHSS score [29, 30], the risk of haemorrhagic transformation secondary to thrombolysis may be greater than the expected benefits for patients whose natural course appears favourable (mRS 0-1). A maximum PPV is therefore required for this threshold since an aggressive therapy may result. In our study where the initial NIHSS was <5 or when the initial deficit regressed within the first 135 minutes, the expected benefit of thrombolysis would have been minimal or nil in 87% and 69% of cases respectively. For initial NIHSS <5, since the gains and risks of thrombolysis are both low, it is difficult to differentiate between the positive effect of thrombolysis and patients' natural outcome. In our study, for these two threshold values (initial NIHSS was <5 or initial deficit regressed within the first 135  minutes), the NPV was acceptable (73% and 76%, respectively). In other words, an initial NIHSS score >5 represented a good prognosis (mRS 0-1) in only 27% of cases, and a neurological deficit that was stable for >135 minutes was also a sign of good prognosis in only 24% of cases. 

The NIHSS thresholds as defined in our study are very similar to those recommended for selecting candidates for thrombolysis [31]. The duration threshold would not be applicable though as it is too close to the cut-off onset-to-needle time of 180 minutes. Furthermore, if symptoms start to regress at 135 minutes, thrombolysis may not be advisable, because in 69% of cases, recovery is likely to be excellent. This may explain the rationale of Albers et al. in the STARS study where patients who were admitted earliest were the last to receive treatment [32]. This raises an important question over the possible benefits of waiting as long as possible before commencing thrombolysis in order to be certain that symptoms are not going to regress, even if national and international guidelines recommend that it be administered as early as possible [31, 33]. Conversely, a less intense set of symptoms lasting for >24 hours does not seem to have a negative prognosis since this type of course is frequently encountered in minor infarctions. Thus, the extent of ischaemic lesions could be approached using a combination of clinical and temporal data and thereby define a gradient such as “NIHSS per minute.” 

## 5. Conclusion

In conclusion, predicting natural course and stroke outcome at the acute phase seems possible. Low (<5) and high (>22) NIHSS cut-off points are effective positive predictive values for good (mRS 0-1) and poor (mRS 4-5 or death) outcomes. Results are less conclusive for intermediate initial NIHSS or for thresholds for symptom duration. In order to stratify decision making, anatomophysiological data resulting from the use of functional MRI techniques (DWI-PWI) must be associated with clinicotemporal data in order to establish precise predictive IS criteria for each individual patient. Indeed some authors have highlighted a higher probability of infarction growth and early neurological deterioration when a mismatch between clinical data and DWI is observed [34, 35]. However, it seems that these data alone cannot identify independent predictors of outcome at 3 months [36, 37]. In order to stratify decision making, clinical and temporal variables should be integrated into the equation alongside neuroimaging data in order to determine natural outcome, and thus the best course of treatment.

## Figures and Tables

**Figure 3:**
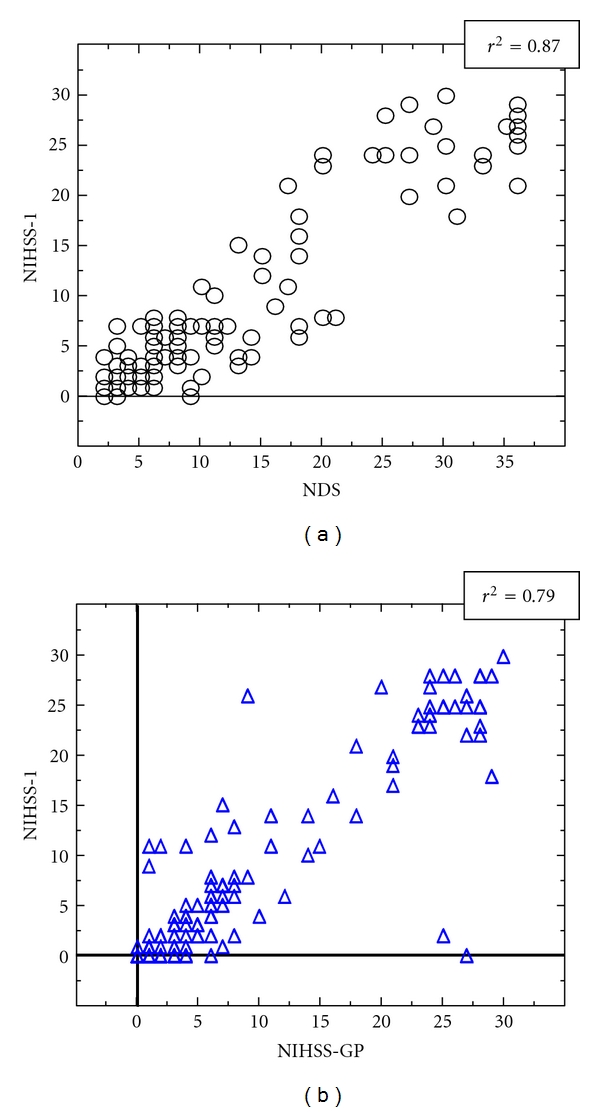
Correlation between NDS and NIHSS-1 (a) and between NIHSS-GP and NIHSS-1 (b).

**Table 1:** Baseline characteristics.

*Medical history*	

Hypertension	55.8%
Cigarette smoking	32.5%
Cardiac dysrhythmia	27.9%
Permanent atrial fibrillation	17.5%
Paroxystic atrial fibrillation	6.7%
Alcohol use	24%
Hyperlipidemia	20.1%
Previous stroke/TIA	15.6%
Arteritis	14.3%
Diabetes mellitus	14.3%
Heart failure	13%
Coronary insufficiency	12.2%
Angina pectoris	6.4%
Myocardial infarction	5.8%

*Infarct territory*	

Total MCA infarction	28 (18.5%)
Total superficial MCA infarction	3 (2%)
Partial superficial MCA infarction	30 (19.9%)
Deep MCA infarction	23 (15.2%)
ACA infarction	2 (1.3%)
Anterior circulation TIA	26 (17.3%)
Localised brain stem infarction	11 (7.3%)
Diffuse brain stem infarction	2 (1.3%)
Thalamic Infarction	6 (4%)
PCA Infarction	4 (2.6%)
Cerebellar infarction	11 (7.3%)
Posterior circulation TIA	10 (6.6%)

*Etiology*	

Atherothrombotic Stroke	
Large artery stenosis >50%	23 (15.3%)
Large artery stenosis <50%	33 (22%)
Microangiopathy	3 (2%)
Cardioembolism	37 (24.7%)
Miscellaneous	4 (2.7%)
Arterial Dissection	1 (0.7%)
Arteritis	1 (0.7%)
Hemopathy/Coagulopathy	3 (2%)
Other	1 (0.7%)
Unknown	48 (31.2%)

*Modified Rankin score at discharge*	

0-1	73 (47.4%)
2	27 (17.5%)
3	10 (6.5%)
4	15 (9.7%)
5	12 (7.8%)
Death	17 (11%)

**Table 2:** Influences of symptom duration and intensity on the course of IS.

	NDS-0 (IC 95%)	NIHSS-GP (IC 95%)	Plateau duration (min.) (IC 95%)
Lesion-free TIA (*n* = 28)	4.3^1^ (3.6–5.1)	2.9^2^ (2.1–3.8)	75.2^3^ (29.6–120.9)
TIA with lesion (*n* = 8)	8.4^1^ (3.6–13.1)	4.7^2^ (2.2–7.3)	442.5^3^ (25.7–859.3)
Infarction (*n* = 118)	14.7^1^ (12.6–16.8)	12.1^2^ (10.1–14.1)	727.5^3^ (609.9–845.1)
mRS 0–3 (*n* = 110)	8^4^ (6.8–9.2)	5.5^5^ (4.3–6.6)	399.1^6^ (296.6–501.6)
mRS 4-5 (*n* = 27)	19.3^4^ (14.2–24.4)	17.0^5^ (12.8–21.2)	931.5^6^ (684.7–1178.2)
Death (*n* = 17)	31.5^4^ (28.2–34.8)	24.1^5^ (20.9–27.3)	1320^6^ (1129.8–1510.2)

**Table 3:** Items assessed in the Neurological Dysfunction Score, reflecting the patient's and/or his/her family's evaluation of symptoms and their variation prior to assessment on admission.

*Motor function*			
	Face	Upper limb	Lower limb

Unknown			
Normal			
Could be used			
Could not be used			
None			

*Sensitivity*			

	Face	Upper limb	Lower limb

Unknown			
Normal			
Slight asymmetry			
Clearly reduced			
Anaesthesia, no feeling at all			

*Cognitive function: speech disturbances, reading writing, calculating difficulties, neglect*			

Unknown			
Normal			
Minimal			
Difficult to understand			
Incomprehensible			

*Posture and gait*			

	Upright position		Gait

Unknown			
Normal			
Possible alone			
Possible with help			
Impossible			

*Limb coordination*			

	Upper limb		Lower limb

Unknown			
Normal			
Slight control			
Very little control			
No control			
